# A hyperechoic mass in femoral vein

**DOI:** 10.1002/ccr3.6552

**Published:** 2022-11-06

**Authors:** Naoko Kaku, Hiroki Sugiyama, Kazufumi Nakamura, Tomoki Furutani, Takashi Hongo, Hiroshi Ito

**Affiliations:** ^1^ Department of Internal Medicine Okayama Saiseikai General Hospital Okayama Japan; ^2^ Department of Cardiovascular Medicine, Faculty of Medicine Dentistry and Pharmaceutical Sciences, Okayama University Okayama Japan; ^3^ Department of Orthopedic surgery Okayama Saiseikai General Hospital Okayama Japan; ^4^ Department of Emergency medicine Okayama Saiseikai General Hospital Okayama Japan; ^5^ Department of Emergency, Critical Care, and Disaster Medicine Okayama University Graduate School of Medicine, Dentistry, and Pharmaceutical Sciences Okayama Japan

**Keywords:** bone fractures, fat embolism syndrome

## Abstract

Here, we present a case of fat embolism syndrome (FES) in which ultrasound sonography and computed tomography successfully visualized fat embolus in the femoral vein. A multimodality approach was particularly useful for early and specific diagnosis.

## Graphical abstract

Ultrasonographic observation of veins was useful for the diagnosis of fat embolism syndrome. The widespread use of ultrasound examination would enable a better understanding of disease progression and a rapid translation into more effective individualized treatment.

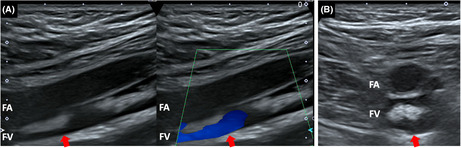

## 1 CASE

**FIGURE 1:**
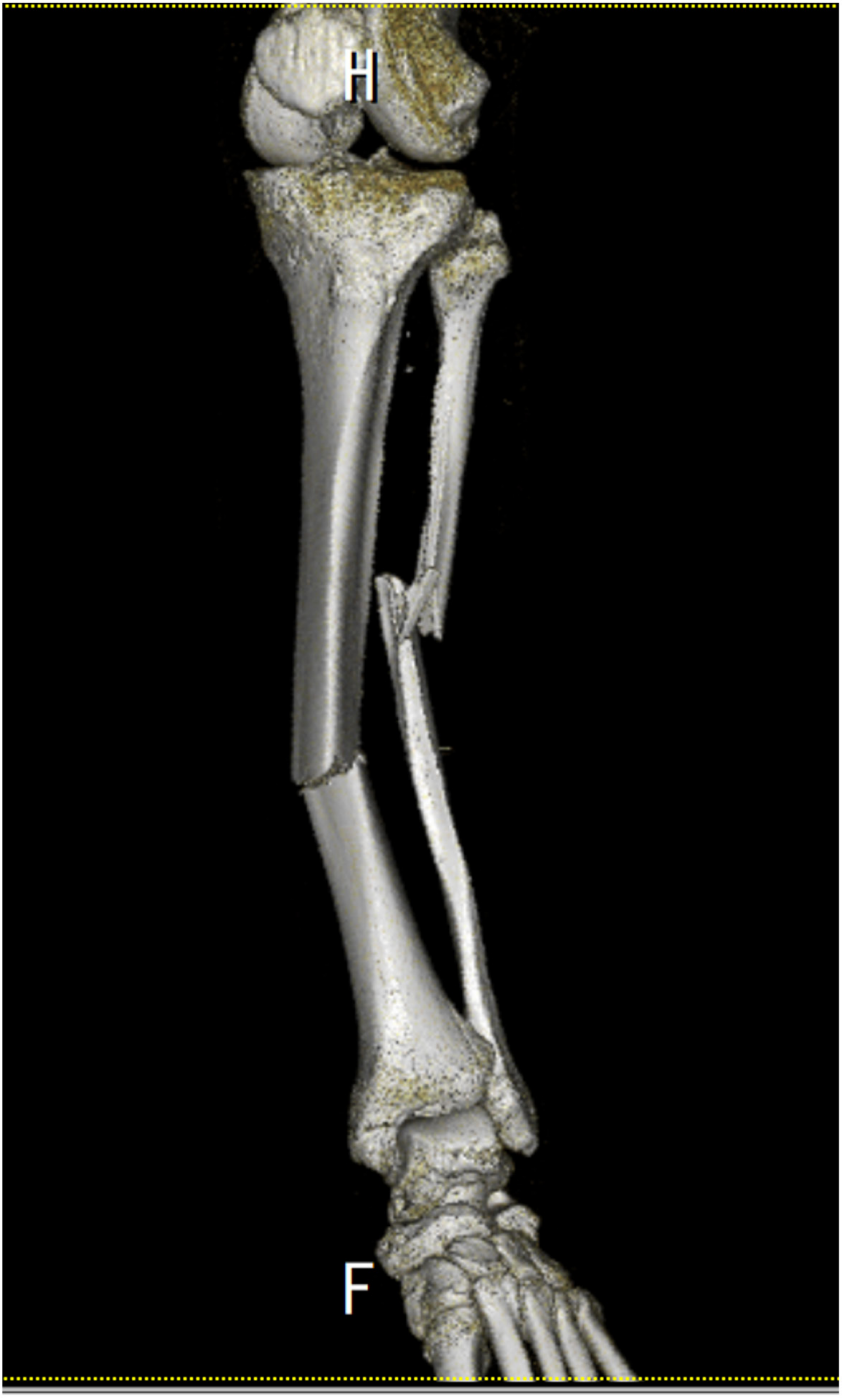
Three‐dimensional volume‐rendered reconstruction of CT images shows the diaphyseal fracture of the left tibia and fibula

**FIGURE 2:**
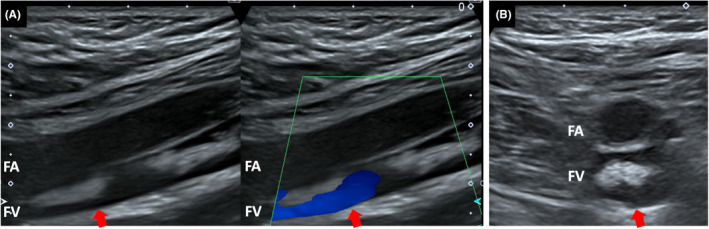
Lower extremity venous ultrasound displayed a soft tissue of 4.6 centimeters in length with high echogenicity, mobility, elasticity, and surrounding blood flow (arrow). A: Long‐axis view (left, B‐mode imaging; right, color Doppler imaging). B: Short‐axis view. FA, femoral artery; FV, femoral vein

A 70‐year‐old man was hospitalized with long‐bone fractures of the left lower limb (Figure 1). Although his baseline vital signs were normal, room air oxygen saturation suddenly decreased to 85% the next day. Transthoracic echocardiography showed a slight increase in tricuspid regurgitation jet velocity (3.0 m/s), but no other findings suggestive of heart failure. There were no abnormal findings in the lung and pulmonary artery on contrast‐enhanced computed tomography (CT). A hyperechoic mass was detected in the left femoral vein by ultrasound sonography (US) (Figure 2A,B).

## 2 QUESTION

What is the cause of respiratory insufficiency?

## 3 ANSWER

**FIGURE 3:**
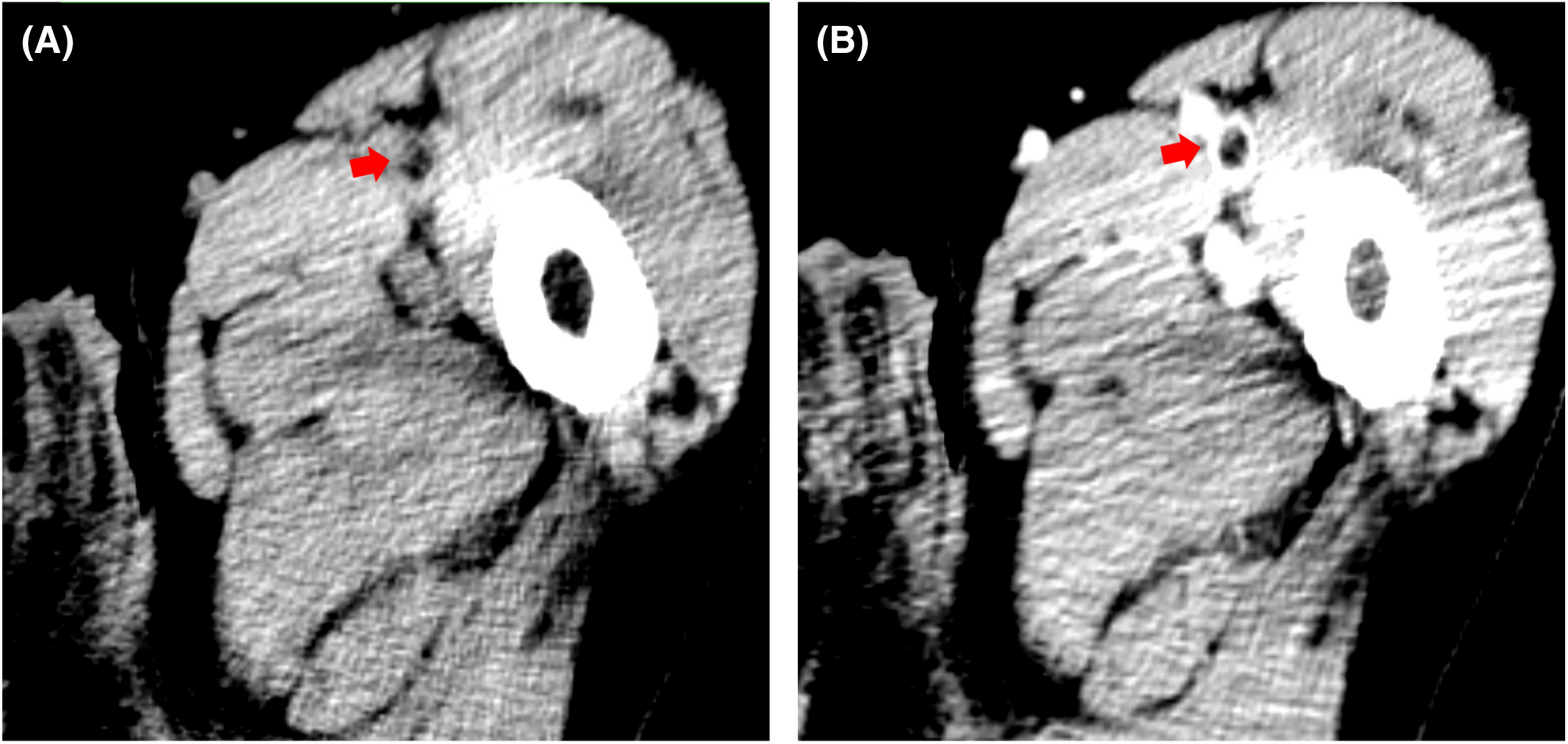
CT documented a filling defect with ‐53HU suggesting fat migration toward the left femoral vein (arrow). A: Nonenhanced image. B: Contrast material‐enhanced image

Large‐sized fat attenuation was observed in the same region on CT (Figure 3A,B); therefore, the patient was diagnosed with fat embolism syndrome (FES).^1^ Serial US revealed that the targeted fat attenuation was regressed at the same site on Day 4 and had disappeared without further embolism episodes on Day 10.

Gurd's and Wilson's diagnosis criteria for FES require at least one major criteria (petechiae, hypoxaemia, central nervous system depression and pulmonary edema) and four minor criteria (tachycardia, pyrexia, emboli present in the retina, fat present in urine, inexplicable drop in hematocrit or platelet values, increasing ESR, and fat globules in the sputum).^2^ In addition, Schonfeld's diagnostic criteria for FES consist of 7 factors (petechiae, X‐ray chest diffuse infiltrates, hypoxemia, fever, tachycardia, tachypnea and confusion). In our case, the only disease‐related feature described in these criteria was hypoxemia. Therefore, our case did not meet these diagnostic criteria. None of the current standard diagnostic criteria are designed to directly detect intravascular fat emboli on imaging.

Previous imaging studies, especially regarding US, that succeeded in documenting a circulating fat embolus are rare.^3^ A unique aspect of FES is that it takes an average of more than 24 h for onset from the time of injury. The clinical convenience of US due to no radiation exposure permits serial time‐course studies, hence contributing to initial diagnosis in the early phase. Because the fat body visualized as a hyperechoic mass on US was nearly indistinguishable from non‐acute thrombosis, it is critical for definitive diagnosis to perform a multimodality approach combining CT examination.

## AUTHOR CONTRIBUTIONS

NK and HS wrote the initial draft of the manuscript. KN and HI was responsible for the drafting and image modification. HS and TF contributed to treat the patient. TH contributed to diagnose and treat the patient. All authors read and approved the final article.

## FUNDING INFORMATION

This report did not receive any specific funding.

## CONFLICT OF INTEREST

None.

## CONSENT

Written informed consent to publish this report was obtained from the patient before the submission process.

## Data Availability

The data that support the findings of this study are available from the corresponding author upon reasonable request.

## References

[1] Hongo T , Naito H , Fujiwara T , Inaba M , Fujisaki N , Nakao A . Incidence and related factors of hypoxia associated with elderly femoral neck fractures in the emergency department setting. Acute Med Surg. 2020;7:e618. doi:10.1002/ams2.618 33364038 PMC7750023

[2] Gurd AR , Wilson RI . The fat embolism syndrome. J Bone Joint Surg Br. 1974;56B:408‐416. 4547466

[3] Burr T , Chaudhry H , Zhang C , Vasilopoulos V , Allam E . Fat embolism in the popliteal vein detected on CT: case report and review of the literature. Radiol Case Rep. 2020;15:2308‐2313. doi:10.1016/j.radcr.2020.09.008 32983305 PMC7494937

